# Toward a documentary standard for performance testing of terrestrial laser scanners used in forensic practice: A statistical procedure to assess change in instrument precision

**DOI:** 10.1111/1556-4029.70256

**Published:** 2026-01-07

**Authors:** Mary Gregg, Bala Muralikrishnan, Meghan Shilling

**Affiliations:** ^1^ Statistical Engineering Division National Institute of Standards and Technology Boulder Colorado USA; ^2^ Sensor Science Division National Institute of Standards and Technology Gaithersburg Maryland USA

**Keywords:** crime scene investigation, crime scene reconstruction, documentary standards, instrument calibration, instrument precision, performance assessment, quality assurance, terrestrial laser scanners

## Abstract

This paper documents efforts by members of the Crime Scene Investigation and Reconstruction (CSIR) subcommittee within the Organization of Scientific Area Committees (OSAC) for Forensic Science, in collaboration with researchers at the National Institute of Standards and Technology, to develop a documentary standard for performance assessment testing of terrestrial laser scanners (TLSs). Intended to be performed by forensic practitioners, this “Interim Performance Assessment” is specifically designed to be inexpensive, concise, and flexible, and is comprised of two parts that separately evaluate instrument accuracy and precision. This paper motivates and details the second of these test procedures, which uses a statistical methodology to assess whether an instrument's point coordinate precision has significantly changed over time. In this paper, the statistical details are reviewed, and the proposed test procedure is illustrated through two examples of longitudinal TLS data. The utility, scope, and limitations of the proposed test procedure are discussed in the context of instrument quality assurance.

**Highlights**

- The paper presents a proposed statistical approach to monitor TLS precision over time.
- The current work uses data collected from two TLS instruments to demonstrate feasibility.
- The proposed test procedure allows forensic practitioners to conduct in‐house quality assurance on TLSs.
- The proposed test procedure is included in a forthcoming OSAC Standard.

## 1 INTRODUCTION

The Crime Scene Investigation and Reconstruction (CSIR) subcommittee within the Organization of Scientific Area Committees (OSAC) for Forensic Science is currently engaged in the development of a terrestrial laser scanner (TLS) Interim Performance Assessment (IPA) for forensic practitioners. The current IPA draft includes two distinct test procedures focused separately on instrument accuracy (“Part I”) and precision (“Part II”). This paper outlines the motivation and challenges behind the IPA development efforts and describes the Part II test procedure assessing instrument precision. A separate manuscript detailing Part I is forthcoming.

Discussions supporting the IPA development began in the summer of 2022 and are currently ongoing. Participants in these discussions include TLS specialists from law enforcement agencies and private firms, and researchers at the National Institute of Standards and Technology (NIST) in the Sensor Science Division (Dimensional Metrology Group) and the Statistical Engineering Division. Multiple data collection efforts have supported the evolution and efficacy of the proposed IPA, with participation from forensic TLS professionals and instrument manufacturers. A report describing these efforts has recently been published [1].

The objectives of this paper are twofold: first, to highlight the challenge of developing a meaningful TLS performance assessment while adhering to the diverse needs of the forensic community; second, to present an accessible overview of one test procedure that meets these requirements. This paper is structured as follows: In Section 2, we motivate the need for the IPA, detail three directives essential to its development, and review terminology and fundamental background information. In Section 3, we align TLS data structures and error sources with the needs of the forensic community to motivate a test procedure that assesses instrument precision over time. In Section 4, we provide an overview of the test procedure, outlining the statistical methodology that offers a formal method of evaluating significant change in instrument precision, and a data visualization technique that enhances the interpretability of the statistical conclusion. In Section 5, we illustrate the test procedure through two examples. In Section 6, we conclude the paper with a discussion.

## 2 BACKGROUND

### 2.1 Motivation and guiding directives

TLSs are portable coordinate measuring instruments whose adoption by law enforcement agencies has fundamentally changed the preservation and documentation of crime and accident scenes [2, 3]. Point cloud data collected by these instruments not only provides a comprehensive 3D visual representation of a scene but also allows retrospective quantitative analysis of the captured point coordinates. Compared to traditional hand tools or laser trackers, TLSs provide a safe, more sophisticated, and more efficient method for obtaining critical scene measurements.

When a measuring instrument provides data that may be used in court, best practice is to perform a periodic quality assurance process that can support the integrity of the instrument's reported values. Most TLSs have recommended calibration intervals defined by their manufacturers. Specific recommendations depend on the manufacturer and instrument model, but annual or biennial intervals are typical. A manufacturer‐performed calibration involves financial investment and requires that a TLS be taken out of service; therefore, having to perform a full, supplemental calibration is an imperfect solution should a question arise about an instrument's integrity within the defined calibration window. Ideally, an “interim” performance assessment test procedure would be available to forensic TLS users that could be performed in‐house, as necessary, to assess instrument performance. Two performance evaluation standards (ASTM E2938‐15 and ASTM E3125‐17) and one field check standard (ISO 17123‐9) are currently published and provide testing procedures to assess TLS operational performance. However, these are industry‐focused documents that require specialized equipment and testing environments that are infeasible for most forensic TLS users. The OSAC CSIR subcommittee addressed this gap by developing an IPA designed to meet the needs and limitations of the forensic community. In their efforts, the subcommittee identified three directives to which test procedures included in the IPA must conform:

1. Inexpensive: Many forensic agencies face budgetary constraints. Test procedures must not require the use of expensive equipment to which most agencies do not have access.
2. Concise: Forensic practitioners have many demands on their time and must accommodate erratic schedules and unexpected interruptions. Test procedures must be capable of being conducted in a reasonable amount of time.
3. Flexible: Agencies may have limited space availability, making dedicated testing facilities and permanent target arrays infeasible. Test procedures must accommodate various indoor environments that may change over time.

By only including test procedures adhering to these directives, the CSIR subcommittee sought to produce an IPA that could be realized by any forensic TLS user, regardless of agency specifics.

### 2.2 Terminology

This section reviews some essential concepts that motivate the specific testing procedure detailed in this manuscript.

#### 2.2.1 Measurement precision

This paper focuses on measurement precision (also simply called “precision”). Following the definition in [4], measurement precision is the closeness of agreement between measured values obtained by replicate measurements on the same or similar objects under specified conditions. In this paper, precision will specifically involve the reproducibility of a set of measured lengths at two significantly different times. We note that precision does not automatically imply accuracy, since measurement values could be quite consistent over time, but still be relatively far from the true value. Nonetheless, a test procedure (like the one here) developed to evaluate an instrument's precision provides useful information about an instrument's operational status.

Precision relates to the spread in values of replicated measurements. Replicated measurements using an instrument with higher (i.e., better) precision will produce measurements closer in value (lower spread) compared to an instrument of lower precision (higher spread). Variance is a statistical measure of the spread in observations, and an instrument's precision can be assessed by estimating the variability in its measurements. Variance estimates are in squared units, however, it's often more convenient to discuss spread in terms of standard deviations. If $\sigma^{2}$ represents the variance of a set of measurements, the standard deviation is defined $s=\sqrt{\sigma^{2}}$. In this paper, we will express—for a single variable—the precision, P, to be the inverse standard deviation, P=1/s. Quantifying the magnitude of a change in precision relative to a change in spread can be done as follows: given a percentage increase in standard deviation, $c_{s}$, the corresponding percentage decrease in precision is $c_{P}=\frac{c_{s}}{c_{s}+1}$.

#### 2.2.2 TLS point coordinates and instrument errors

TLSs capture data as clouds of Cartesian coordinate points defined by the instrument's coordinate system. That is, the location of any particular point is represented as $\left(X,Y,Z\right)$, where X, Y, and Z are units of length as measured from the location of the instrument when it performed the scan, where XY is the horizontal plane and Z is coincident with the vertical rotation angle of the scanner [5]. Alternatively, these points may be represented as spherical coordinates $\left(\theta ,\varphi ,r\right)$, where θ is the azimuth angle, φ is the elevation angle, and r is the ranging distance. The conversion from Cartesian to spherical coordinates is given in Table 1. When referring to instrument performance, spherical coordinates are advantageous over Cartesian coordinates as opto‐mechanical misalignments in the TLS system manifest as angular and ranging errors through known relationships [5, 6].

**TABLE 1:** Relationship between Cartesian coordinates $\left(X,Y,Z\right)$ and spherical coordinates $\left(\theta ,\varphi ,r\right)$.

Variable	Description	Relationship to Cartesian coordinates
r	Ranging value	$r=\sqrt{X^{2}+Y^{2}+Z^{2}}$
θ	Azimuth angle	$\theta =\arctan\left(\frac{Y}{X}\right)$
φ	Elevation angle	$\varphi =\text{arcsine}\left(\frac{Z}{r}\right)$

*Note*: The ranging value, r, has units corresponding to the Cartesian coordinates. The calculated angular measurements will be in radians.

#### 2.2.3 Variance for multiple variables: covariance matrices

TLSs capture 3D spherical coordinates, meaning there are three variables, $\left(\theta ,\varphi ,r\right)$, recorded for each point coordinate. Each variable has its own variance. Additionally, each variable has a relationship (correlation) with every other variable. The statistical measure for the relationship between a pair of variables is termed the “covariance.” The overall variability and, by association, precision, of a TLS therefore relates to six components: the variance in the angular and range coordinates, $\sigma_{\theta }^{2},\sigma_{\varphi }^{2},\sigma_{r}^{2}$, and the three covariance terms between each pair of variables, $\sigma_{\theta \varphi },\sigma_{\mathrm{\theta r}},\sigma_{\mathrm{\varphi r}}$. For mathematical convenience, these terms are collected in a variance–covariance matrix, commonly referred to as the “covariance matrix”:

$$\sum =\left[\begin{array}{ccc} \sigma_{\theta }^{2} & \sigma_{\theta \varphi } & \sigma_{\mathrm{\theta r}}\\ \sigma_{\theta \varphi } & \sigma_{\varphi }^{2} & \sigma_{\mathrm{\varphi r}}\\ \sigma_{\mathrm{\theta r}} & \sigma_{\mathrm{\varphi r}} & \sigma_{r}^{2} \end{array}\right]$$

Similar to variance and standard deviation, it is often easier to interpret covariance terms through correlation. The correlation between two variables is their covariance divided by their standard deviations, for example, the correlation between θ and φ is $\rho_{\theta \varphi }=\frac{\sigma_{\theta \varphi }}{\sqrt{\sigma_{\theta }^{2}}\;\sqrt{\sigma_{\varphi }^{2}}}$. Correlation is a unit‐less value between $\left[-1,1\right]$ whose sign relates to the directionality (positive or negative) of the relationship between the two variables and the magnitude relates to the strength of the relationship.

## 3 APPROACH

The current draft IPA includes two parts, with Part I related to instrument accuracy and Part II related to instrument precision. This paper focuses on the second part, which assesses whether an instrument's precision has changed between two timepoints.

The typical TLS user is interested not in the point coordinates themselves, but in dimensional information derived from the coordinates (e.g., the height of a door, the distance between two objects, the volume of a room). It is natural, then, to think that a test procedure assessing instrument performance would evaluate the instrument's accuracy of the point‐to‐point lengths, as those are the measurands of interest and accuracy is the first fundamental property of appropriate instrument behavior. Indeed, such testing procedures are included in Part I. However, the Part I assessment is limited in scope to remain in compliance with the directives listed in Section 2.1 Additionally, dimensional measurements are downstream from the primary point coordinates collected by the instrument. The accuracy and precision of the lengths derived from the point coordinates will be dependent on the accuracy and precision of the point coordinates themselves. Furthermore, TLS errors due to opto‐mechanical misalignments have a well‐understood relationship to spherical coordinates. Therefore, to provide a more comprehensive assessment of instrument performance, an additional testing procedure, Part II, is included in the IPA that evaluates an instrument's spherical coordinate precision as measured by the covariance matrix.

It is impractical to define a threshold for appropriate TLS precision. First, appropriate performance will vary across different models of instruments. Second, manufacturers do not typically provide performance specification values corresponding to the parameters in the covariance matrix, ∑. Third, even if such values were available, they would only be representative of instrument performance under specific conditions in highly controlled environments and would not represent appropriate performance in practice. As a result, rather than comparing instrument performance against a threshold, the testing procedure described here monitors an instrument's precision over time.

## 4 TEST PROCEDURE

The overview of the test procedure is straightforward: Let $\sum_{1}$ be the covariance matrix representing the instrument's overall angular and ranging variability at some baseline state and let $\sum_{2}$ be the covariance matrix representing the instrument's overall variability at some later point in time. We will refer to these two timepoints as “Baseline” and “Testing,” respectively. Measurements are collected at the Baseline time point to establish an estimate of $\sum_{1}$. Later, at the testing timepoint, additional measurements are collected to estimate $\sum_{2}$. A statistical procedure [7] is applied to the collected data to test the null hypothesis $\sum_{1}=\sum_{2}$. If the statistical procedure provides sufficient evidence to reject the null hypothesis, the user can conclude that there has been a significant change in the instrument's operational state and should consider the implications of this as discussed briefly in Section 6 of this paper. Alternatively, the test procedure may indicate a lack of evidence to reject the null hypothesis. In such cases, it is important to note that the user cannot definitely conclude from the statistical procedure that there has been no change in precision, as lack of evidence for a change is not equivalent to strong evidence that no change has occurred. However, in the case that the user fails to reject the null hypothesis, they may then reasonably consider this lack of evidence in conjunction with the data visualization technique described below to make an experience‐based judgment about whether the instrument remains in the approximate operational state it was in at the Baseline timepoint.

### 4.1 Spherical residuals and estimation of ∑

To estimate ∑ at a given timepoint, a stationary target array is scanned from multiple TLS positions. Let T represent the number of targets in the array and let P denote the number of scanning positions. Let $\left(x_{\mathrm{tp}},y_{\mathrm{tp}},z_{\mathrm{tp}}\right)$ denote the Cartesian coordinates of the *t*th target measured from the *p*th TLS position, t=1,…,T;p=1,…,P.

Once data are collected, a series of transformations are performed before ∑ can be estimated.

1. Convert to a centralized frame of reference. The data is transformed to a centralized frame of reference using a simplified version of a bundle adjustment process [8, 9]. This process operates by iteratively solving for the rigid body transformation translation and rotation parameters that minimize the difference between the individual coordinates and average coordinates across all positions. Let $\left(x_{\mathrm{tp}}^{\prime },y_{\mathrm{tp}}^{\prime },z_{\mathrm{tp}}^{\prime }\right)$ define the Cartesian coordinates of the *t*th target measured from the *p*th TLS position converted to this centralized frame of reference.
2. Obtain composite coordinates. Composite coordinates are obtained in the centralized frame of reference by averaging the P replicates of each target. Define the centralized composite coordinates as $\left({\bar{x}}_{t}^{\prime },{\bar{y}}_{t}^{\prime },z_{t}^{\prime }\right)$, where $\left({\bar{x}}_{t}^{\prime },{\bar{y}}_{t}^{\prime },z_{t}^{\prime }\right)=\left(\frac{1}{P}\sum_{p=1}^{P}x_{\mathrm{tp}}^{\prime },\frac{1}{P}\sum_{p=1}^{P}y_{\mathrm{tp}}^{\prime },\frac{1}{P}\sum_{p=1}^{P}z_{\mathrm{tp}}^{\prime }\right)$.
3. Convert composite coordinates back to the individual frames of reference. The composite coordinates are back‐transformed to the original frames of reference using the parameters obtained in Step 1. Let $\left({\bar{x}}_{\mathrm{tp}},{\bar{y}}_{\mathrm{tp}},{\bar{z}}_{\mathrm{tp}}\right)$ denote the composite Cartesian coordinates for target t transformed back to the *p*th frame of reference.
4. Calculate the spherical residuals. Let $\left(\theta_{\mathrm{tp}},\varphi_{\mathrm{tp}},r_{\mathrm{tp}}\right)$ be the spherical coordinates corresponding to Cartesian coordinates $\left(x_{\mathrm{tp}},y_{\mathrm{tp}},z_{\mathrm{tp}}\right)$ and $\left({\bar{\theta }}_{\mathrm{tp}},{\bar{\varphi }}_{\mathrm{tp}},{\bar{r}}_{\mathrm{tp}}\right)$ be the spherical coordinates corresponding to $\left({\bar{x}}_{\mathrm{tp}},{\bar{y}}_{\mathrm{tp}},{\bar{z}}_{\mathrm{tp}}\right)$. The spherical residuals for the *t*th target at the *p*th position are defined as $\left(e_{\mathrm{tp}}^{\left(\theta \right)},e_{\mathrm{tp}}^{\left(\varphi \right)},e_{\mathrm{tp}}^{\left(r\right)}\right)=\left(\theta_{\mathrm{tp}}-{\bar{\theta }}_{\mathrm{tp}},\varphi_{\mathrm{tp}}-{\bar{\varphi }}_{\mathrm{tp}},r_{\mathrm{tp}}-{\bar{r}}_{\mathrm{tp}}\right)$

Once the spherical residuals have been calculated, ∑ can be estimated. Define X to be the matrix of spherical residuals. As T targets were measured from each of P positions, there are a total of N=T×P sets of spherical residuals, so X has dimension N × 3. The estimate of ∑ is then calculated as

$$\hat{\sum }=\frac{1}{N-1}{\left(X-1\bar{x}\right)}^{T}\left(X-1\bar{x}\right)$$

where 1 is a length‐N column vector of ones and $\bar{x}$ is the row vector of column means of X.

### 4.2 Statistical procedure

To perform the hypothesis test $H_{0}:\sum_{1}=\sum_{2}$, data are collected at two timepoints and the spherical residuals are calculated according to the section above. The applied statistical procedure comes from the Robust Omnibus Test in O'Brien (1992) [7], which is a multivariate analog to Levene's test. We summarize the calculations here.

Let $e_{\mathrm{ijk}}$ represent the residual on the *k*th spherical coordinate (k=1,2,3, corresponding to θ,φ, and r, respectively) on the *j*th set of residuals ($j=1,\ldots ,N_{i}$) calculated at timepoint i (i=1,2). For $k^{\prime }=k,\ldots ,K$, define the following values:

$$M_{\mathrm{ik}}=\text{median of variable}\;k\;\mathrm{at}\;\text{timepoint}\;i,$$

$$Z_{{\text{ijkk}}^{\prime }}=\left(e_{\mathrm{ijk}}-M_{\mathrm{ik}}\right)\left(e_{{\mathrm{ijk}}^{\prime }}-M_{{\mathrm{ik}}^{\prime }}\right),$$

$$W_{{\text{ijkk}}^{\prime }}=\frac{Z_{{\text{ijkk}}^{\prime }}}{{\left|Z_{{\text{ijkk}}^{\prime }}\right|}^{1/2}}=\mathrm{sign}\left(Z_{{\text{ijkk}}^{\prime }}\right)\left({\left|Z_{{\text{ijkk}}^{\prime }}\right|}^{\frac{1}{2}}\right),$$

$$W_{\mathrm{ij}}=\text{the vector of}\;W\;\text{values from the}\;j\mathrm{th}\;\mathrm{set}\;\text{of residuals}\;\mathrm{at}\;\text{the}\;i\mathrm{th}\;\text{timepoint}.$$

The statistical procedure then comprises a one‐way analysis of variance analysis comparing $W_{1j}$ and $W_{2j}$ values, which reduces to Hotelling's $T^{2}$ test as I=2.

The statistical procedure produces a test statistic and a corresponding probability (“*p*‐value”) of the observed data assuming that the null hypothesis is true. If the probability is high, then the observed data are consistent with what would be expected if the instrument's precision has not changed. However, if the probability is low—specifically, less than some significance threshold, α—the user has evidence to believe that the instrument's precision has significantly changed. The user's confidence in rejecting the null hypothesis (determining the instrument state has changed) is 1−α, meaning that confidence increases as the threshold decreases. It is up to the user to set their desired significance threshold, though typical values for α are 0.1, 0.05, and 0.01. A higher α value makes it easier to catch scanners whose precision has changed but increases the chance of erroneously concluding a scanner's performance has changed when it actually has not. A stringent threshold of 0.01 (1%) means the evidence must be very strong for a user to conclude that an instrument's precision at the time of testing is significantly different than it was when the instrument was in the baseline state but allows the user to be 99% confident in their conclusion that the instrument has changed state. A more liberal threshold of 0.1 (10%) requires less evidence to conclude a state change, potentially catching more “compromised” scanners, but the user will only be 90% confident in their conclusion. For the examples presented in this paper, we use a significance threshold of α=0.01.

### 4.3 Visualizing differences in covariance matrices through data ellipses

If the statistical procedure provides evidence that an instrument's precision has changed, the user might then want to know in what way the precision has changed. Recall that the overall variance of the instrument is related to six components consisting of the variability in the individual angular and ranging values and their pairwise correlations. A significant change in precision could be induced by a change in any one (or combination of) these six parameters. While comparing individual numerical values across the estimates of $\sum_{1}$ and $\sum_{2}$ can give a general idea of what has changed, a visual summary will aid interpretation. Therefore, we recommend pairing the statistical hypothesis test with the visualization technique of Friendly et al. (2013) [10] and Friendly and Sigal (2020) [11]. This technique summarizes the respective spreads and relationship between a pair of variables through an ellipse whose boundary is proportional to the variance and covariance estimates from those variables. By drawing such ellipses for the pairings of θ,φ and r, the user can compare the size and shape of ellipses between the two timepoints to easily visualize differences in the instrument's precision.

### 4.4 A note on data collection requirements

**FIGURE 1:**
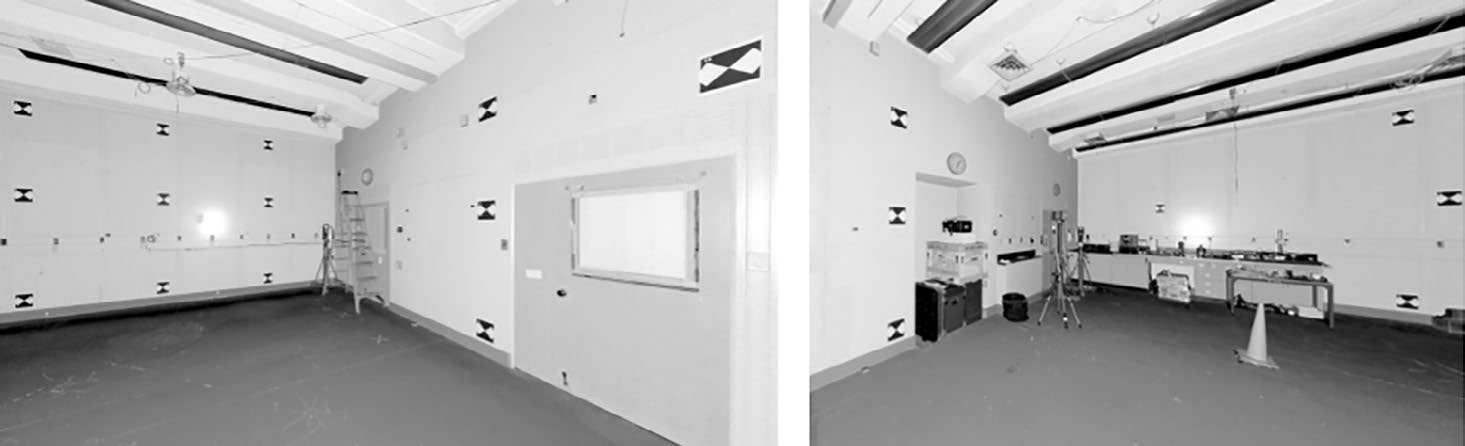
An example array of 20 checkerboard targets distributed across three walls. Left panel: Thirteen targets are on walls 1 and 2; Right panel: Seven targets are on walls 2 and 3.

To estimate the instrument's precision at a given timepoint, it is necessary to perform multiple scans of a stationary target array. The current IPA draft suggests collecting Cartesian coordinates from 20 checkerboard targets from four TLS scan positions at both the Baseline and Testing timepoints. An illustration of such a target array is presented in Figure 1. The proposed 20 targets and four positions have been found to work well in practice, but these specifications are not absolute and may change depending on other limitations, for example, room size. Target placement and TLS scanning positions should be chosen such that a range of horizontal and vertical angles are sampled, while maintaining appropriate angles of incidence. Specific instructions on target and scanning locations will be provided in the published documentary standard. Stability of targets within a timepoint is required, but stability is not assumed across the two timepoints. That is, the 20 targets cannot be rearranged between the four scans conducted at a given timepoint, but the placement of targets at the Testing timepoint may be different than they were at the Baseline timepoint. This flexibility conforms to the test procedure in Directive 3 in Section 2.1, allowing agencies to utilize space as available.

## 5 EXAMPLES

In this section, we illustrate the use of the statistical procedure and the accompanying data visualization technique through two examples. The statistical method operates in terms of variance and covariance, but we will interpret these terms through standard deviations and correlations. The data presented in the first example are from a short‐range, high precision TLS, while the data used in the second example were collected from a TLS equipped with an automatic, user‐initiated adjustment procedure. This adjustment improves the accuracy of the scanner by updating the internal TLS error model that automatically corrects for some opto‐mechanical misalignments. All example datasets were collected in the large‐scale dimensional metrology laboratory at NIST and consist of coordinate measurements from fixed arrays of 20 targets using four instrument scanning positions.

### 5.1 Example 1: Precision degradation over time

In this example, the Baseline data are measurements collected in 2020 and the Testing data are measurements collected in 2025. Details on these data collections can be found in [1, 9], respectively. The TLS did not undergo any manufacturer‐performed calibration in the five years separating the two data collections. Using the statistical process outlined above, we assess whether the instrument's precision has significantly changed between 2020 and 2025.

Applying the Robust Omnibus Test produces a test statistic of 8.75, which has a corresponding p‐value less than 0.0001. Since the p‐value is less than 0.01, we reject the null hypothesis and conclude that there has been a significant change in the instrument's precision from 2020 to 2025. For a more nuanced understanding of specific changes, we can compare individual components in the estimated covariance matrices shown below (where angular values are reported in arcseconds, and ranging values are reported in mm).

$${\hat{\sum }}_{2020}=\left[\begin{array}{ccc} 12.84 & -1.20 & 0.05\\ -1.20 & 2.52 & 0.00\\ 0.05 & 0.00 & 0.004 \end{array}\right],{\hat{\sum }}_{2025}=\left[\begin{array}{ccc} 18.06 & 6.10 & -0.06\\ 6.10 & 44.76 & -0.07\\ -0.06 & -0.07 & 0.01 \end{array}\right]$$

Comparing the diagonal elements in the two matrices, the variability has increased in all three spherical coordinates. However, the most notable change is in the estimate of $\sigma_{\varphi }^{2}$. In 2020, the standard deviation in φ was estimated to be $\sqrt{2.52}=1.59$; in 2025, this increased to about $\sqrt{44.76}=6.69$. This is a 320% increase in spread, which corresponds to an approximate 76% decrease in precision. In contrast, θ and r have decreased in precision by 15% and 40%, respectively.

**FIGURE 2:**
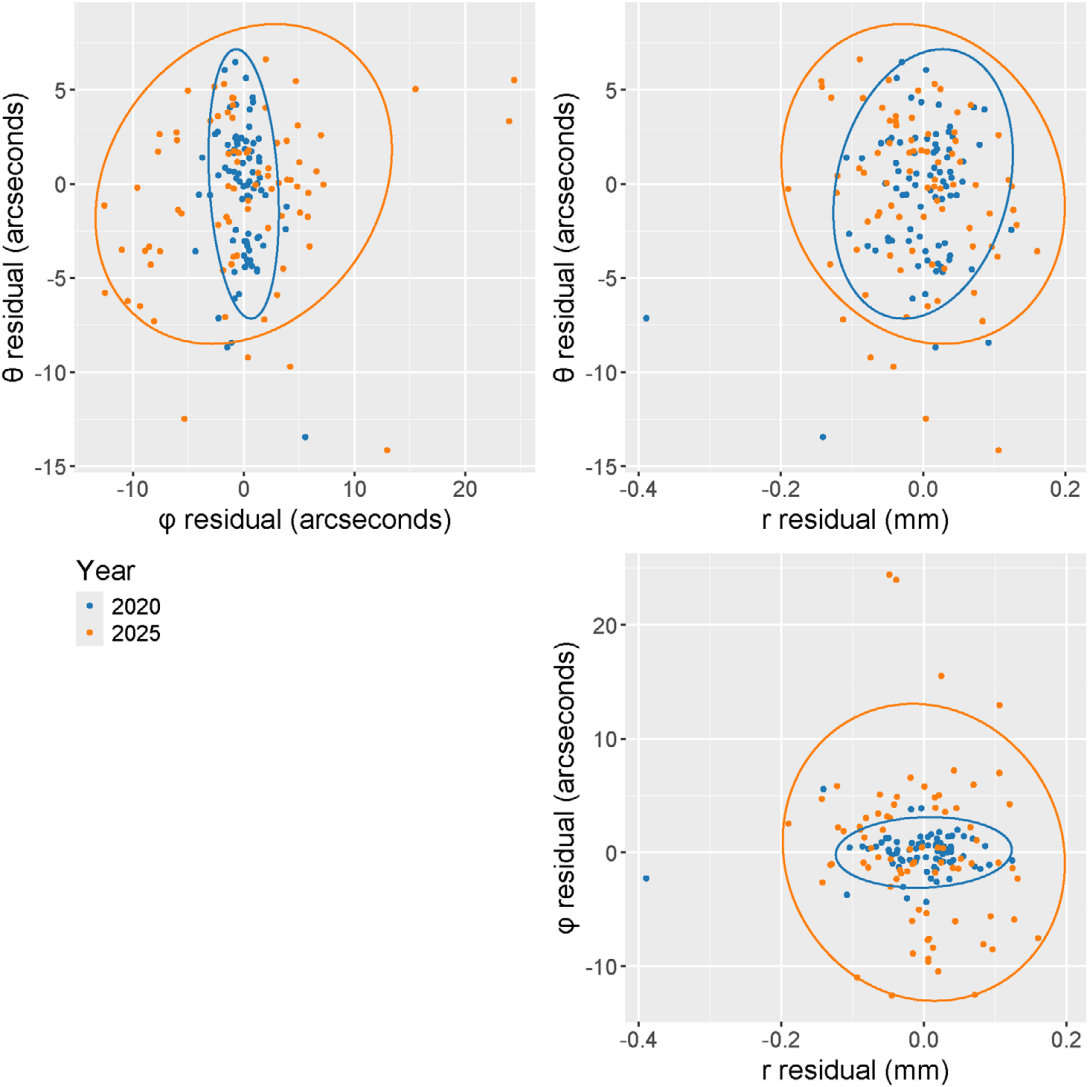
Scatterplots of spherical residuals from data collected on a short‐range, high‐precision TLS in 2020 (blue) and in 2025 (orange). Top left panel: θ vs. φ; Top right panel: θ vs. r; Bottom right panel: φ vs. r. Data ellipses according to the methods of [10, 11] have been added to visualize the estimated covariance matrices.

Figure 2 illustrates the change in angular and ranging precision of the TLS at the two time points by plotting the spherical coordinate residuals. The three panels in Figure 2 correspond to the three variable pairings, with θ vs. φ in the top left, θ vs. r in the top right, and φ vs. r in the bottom right. The third panel in Figure 2 has purposefully been aligned below, rather than adjacent to, the second panel, to highlight the correspondence in the horizontal axis. Ellipses proportional to the corresponding variances and covariances have been added to these scatterplots according to the methods of Friendly et al. [10] and Friendly and Sigal [11]. A simple way to visualize and interpret the differences in the covariance matrices is by comparing the size and shape of these ellipsoids. The size of an ellipse in the horizontal and vertical directions relates to the variability in the corresponding variable, while the shape of the ellipse relates to the correlation between the variables. In each panel of Figure 2, the blue ellipse (corresponding to 2020) is encircled by the orange ellipse (corresponding to 2025), illustrating how the overall precision of the instrument has degraded. The most notable differences between the orange and blue ellipses are in the horizontal direction in the upper left panel and the vertical direction in the lower right panel. These correspond to the large change in the estimate of $\sigma_{\varphi }^{2}$, and it is easy to see from either of these panels that the instrument's elevation angle measurements were significantly more precise in 2020 than they were in 2025.

### 5.2 Example 2: Pre‐ and post‐automatic adjustment procedure

In this example, the Baseline and Testing measurements were collected within two hours of each other from the same array of targets, the setup of which is described in [1]. The Baseline measurements were obtained from the TLS prior to the integrated automatic adjustment procedure being initiated. After collecting the Baseline measurements, the automatic adjustment procedure was performed and a second set of measurements that represent the Testing data was collected.

**FIGURE 3:**
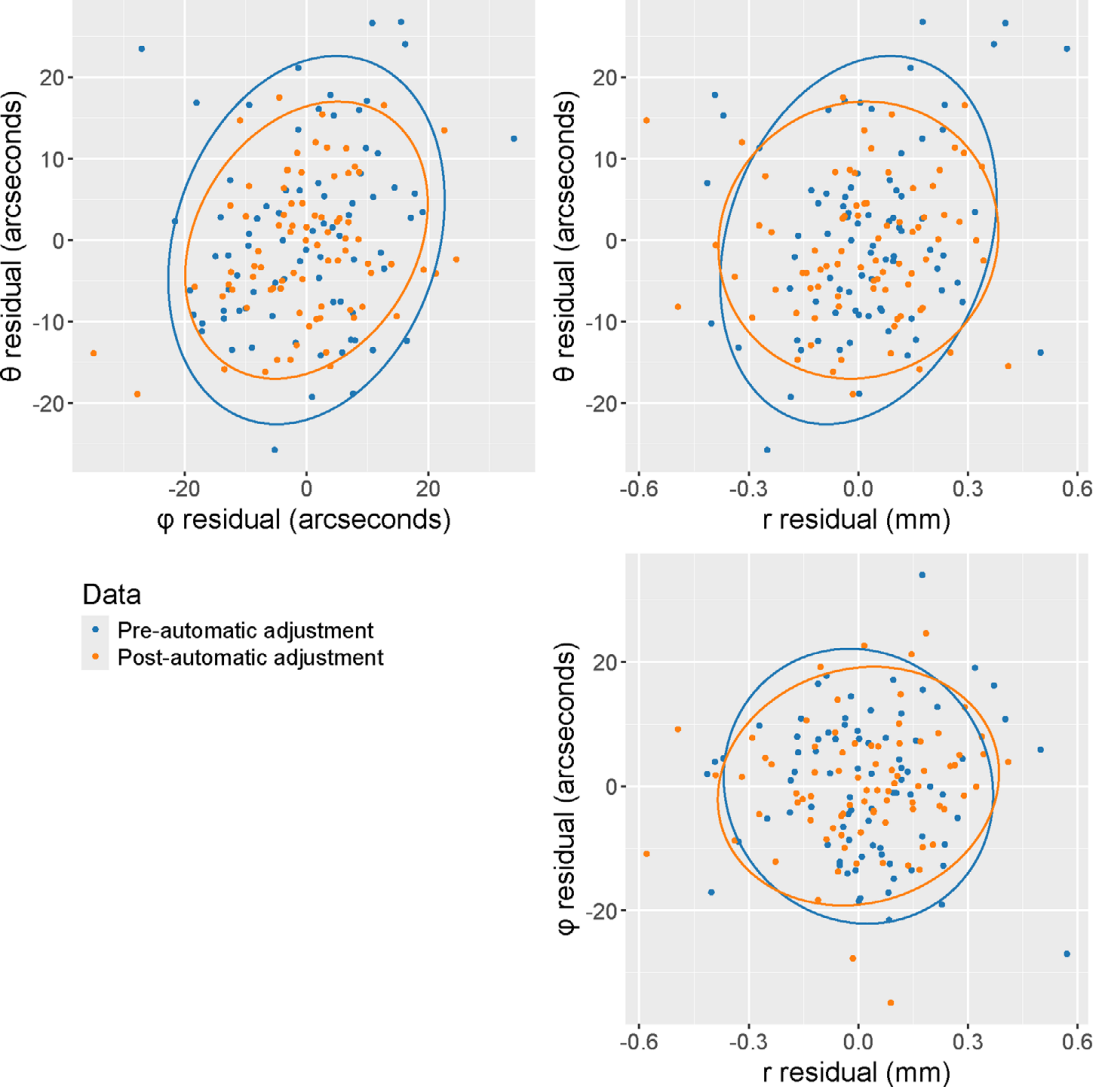
Scatterplots of spherical residuals from data collected on a TLS equipped with an automatic, user‐initiated adjustment procedure. Blue points correspond to data collected prior to the adjustment procedure being initiated; orange points correspond to data collected post‐adjustment. Bottom right panel: φ vs. r. Data ellipses according to the methods of [10, 11] have been added to visualize the estimated covariance matrices.

Applying the Robust Omnibus Test produces a test statistic of 1.69, and a corresponding *p*‐value of 0.127. The *p*‐value is larger than 0.01, so there is insufficient evidence to conclude that the instrument's precision has significantly changed from baseline to two hours later. With angular measurements in arcseconds and ranging measurements in mm, the estimated covariance matrices from the two sets of data are shown below, and the corresponding data ellipses are shown in Figure 3.

$${\hat{\sum }}_{\mathrm{pre}}=\left[\begin{array}{ccc} 127.65 & 27.71 & 0.50\\ 27.71 & 128.58 & -0.03\\ 0.50 & -0.03 & 0.04 \end{array}\right],{\hat{\sum }}_{\text{post}}=\left[\begin{array}{ccc} 72.27 & 21.95 & 0.09\\ 21.95 & 99.02 & 0.23\\ 0.09 & 0.23 & 0.04 \end{array}\right]$$

In the top left panel of Figure 3, the orange ellipse (post‐automatic adjustment) is slightly smaller than the blue ellipse (pre‐automatic adjustment), corresponding to the slight decrease in the angular variance terms seen in ${\hat{\sum }}_{\text{post}}$ compared to ${\hat{\sum }}_{\mathrm{pre}}$. The correlation between θ and r also appears nominally lessened after the automatic adjustment procedure, as the orange ellipse (post‐adjustment) in the top right panel of Figure 3 exhibits less elongation (“stretch”) compared to the blue ellipse (pre‐adjustment). This is seen in the decrease in the estimates of $\rho_{\mathrm{\theta r}}$, with a pre‐adjustment estimate of $\frac{0.50}{\sqrt{127.65}\sqrt{0.04}}=0.22$ and a post‐adjustment estimate $\frac{0.09}{\sqrt{72.27}\sqrt{0.04}}=0.05$. However, such differences are minimal, and the overlap in residuals and general similarity displayed between the two ellipses across the three panels in Figure 3 supports the conclusion drawn by the statistical hypothesis test that the overall instrument precision has not changed significantly.

Initially, this conclusion may be surprising. The adjustment procedure is designed to improve instrument performance and some marginal improvement is evident in Figure 3. Why did the statistical procedure not detect this change? The answer is that only large differences in precision will provide sufficient evidence to support the conclusion that instrument performance has changed. This is a practical characteristic, as this test procedure is not intended to affirm that some procedure has improved performance, but in practice will be used to monitor for changes in instrument precision large enough to be of concern.

## 6 DISCUSSION

This paper summarizes the ongoing efforts of the OSAC CSIR subcommittee to develop an “Interim Performance Assessment” suitable for forensic TLS users and describes one test procedure included in the IPA that compares TLS precision between two timepoints. This test procedure is achieved without the use of external reference artifacts and may be suited for forensic TLS users who must attest to the validity of evidence presented in court but may have limited resources to invest in quality assurance procedures. Using established statistical methodology, the test procedure compares the instrument's point coordinate variability at an initial timepoint (Baseline) to the instrument's point coordinate variability at a later timepoint (Testing), providing the user with a principled method for assessing if the instrument's performance has significantly changed over time.

The test procedure presented in this manuscript is not intended to replace periodic manufacturer‐performed calibrations. Instead, it is presented as an intermediate solution for assessing instrument performance between regularly scheduled maintenance services. In fact, this interim testing procedure is strengthened by association with manufacturer‐performed calibrations. While the Baseline instrument state could be any point in time, an optimal point at which to establish Baseline performance is after receiving the instrument back from a manufacturer‐performed calibration, when confidence in the instrument's performance is highest. Evaluating whether a notable change in precision has occurred since this initial condition may be informative as to the current operational state of the instrument.

A change in an instrument's precision could manifest in various ways. Moreover, without a value defining acceptable precision, it is infeasible to define a threshold for precision change that corresponds to unacceptable instrument performance. The statistical procedure in this paper only tests for a statistically significant change in precision, which does not necessarily equate to a test of appropriate operational condition. Therefore, in addition to performing the statistical procedure, it is recommended that users visualize the estimated covariance matrices through data ellipses. This data visualization technique gives the user a simple, interpretable way to compare the consistency of the instrument at the two timepoints and provides nuance to the binary decision threshold inherent in a statistical hypothesis test. If a significant change in precision is found by this test, the user must then decide what action, if any, should be taken. The range of possible actions spans from doing nothing to the costly option of having the instrument professionally serviced. The appropriate action will depend on the comprehensive (Part I and Part II) IPA results, as well as the performance needs of the instrument. Specific guidance on this decision will be provided in the forthcoming standard. Recognizing the computational burden of this proposed Part II procedure, we are in the process of developing a web application tool that will perform the required calculations for both the statistical test and the data visualization. This tool will be provided to users free of charge to facilitate the future adoption of the IPA standard.

## FUNDING INFORMATION

This work was supported with funding from the NIST Special Programs Office.

## CONFLICT OF INTEREST STATEMENT

There are no conflicts of interest to report.

## DISCLAIMER

Commercial equipment and materials may be identified in order to adequately specify certain procedures. In no case does such identification imply a recommendation or endorsement by the National Institute of Standards and Technology (NIST), nor does it imply that the materials or equipment identified are necessarily the best available for the purpose. Contributions from staff of NIST, an agency of the US Government, are not subject to copyright in the United States.

## Data Availability

The data that support the findings of this study are available from the corresponding author upon reasonable request.
